# Gender-sensitive and intersectionality-informed health indicators for health reporting: a scoping review protocol

**DOI:** 10.1136/bmjopen-2024-091549

**Published:** 2024-11-09

**Authors:** Hande Gencer, Anke-Christine Saß, Franziska Prütz

**Affiliations:** 1 Department of Epidemiology and Health Monitoring, Robert Koch Institute, Berlin, Germany

**Keywords:** PUBLIC HEALTH, Methods, Health Equity, Review, SOCIAL MEDICINE

## Abstract

**Abstract:**

**Introduction:**

Gender is a well-established social determinant of health and health (in)equality. Gender-sensitive health indicators for health monitoring and health reporting can support gender mainstreaming in relevant policy areas and inform strategies to promote gender equality. They generally lack theoretical approaches to explain gender inequalities in health in the context of individual, social and structural circumstances. Gender-sensitive and intersectionality-informed health indicators provide a more accurate and nuanced picture of health outcomes and risks but are often lacking. The aim of this scoping review is to map the evidence on gender-sensitive and intersectionality-informed indicators for health reporting in order to support the development of an indicator set for German Federal Health Reporting.

**Methods and analysis:**

This scoping review follows Arksey and O’Malley’s methodological framework and its extension by Levac *et al* and will be reported according to the Preferred Reporting Items for Systematic Reviews and Meta-Analyses extension for Scoping Reviews. We will conduct a database search complemented by a backward citation search using English search terms to find research articles and grey literature (eg, reports, policy/working papers, book chapters) reporting on gender-sensitive health indicators in the context of health reporting in the European Union-27 and Organisation for Economic Cooperation and Development published since 2014. Electronic databases include Medline, PsycINFO, Embase, Scopus, CINAHL and Cochrane Library. Other resources include targeted searches on websites relevant to national and international health reporting and a Google Search to include further eligible literature. After removing duplicates, two reviewers will independently screen all titles/abstracts and full texts for eligibility for inclusion and extract the data from included articles using a data extraction form. The results will be synthesised both narratively and descriptively and, where appropriate, presented in tables and graphs.

**Ethics and dissemination:**

No ethical approval is required for this study. Findings will be disseminated through peer-reviewed publication, conference presentations and meetings with relevant stakeholders in health monitoring and reporting.

**STRENGTHS AND LIMITATIONS OF THIS STUDY**

- This scoping review protocol follows the Preferred Reporting Items for Systematic Review and Meta-Analysis Protocols (PRISMA) guidelines for reporting systematic review protocols.
- This scoping review will employ rigorous methodological frameworks (Arksey and O’Malley and Levac *et al*) and the latest reporting guidelines (PRISMA extension for Scoping Reviews) for scoping reviews.
- We will apply a broad search strategy with wide inclusion criteria and no language limitations.
- The restriction to the settings of European Union-27 and Organisation for Economic Cooperation and Development member states may introduce a bias towards wealthier countries with more developed healthcare systems and overlook global applicability.

## Introduction

 Gender alongside other social and environmental factors is a well-established determinant of health.^1 2^ It is one of the main elements of the WHO’s Core Social Determinants of Health framework.^3^ Gender equality in health and well-being is an important component of the United Nations’ (UN) Sustainable Development Goals.^4^ Several international organisations have set themselves the task of routinely measuring gender (in)equality in various countries, including the Organisation for Economic Cooperation and Development’s (OECD) Social Institutions and Gender Index,^5^ the UN Development Programme’s Gender Inequality Index^6^ and the Gender Equality Index for the European Union (EU).^7^

Monitoring and reporting on gender equality in the form of continuously provided health data is of great importance for public health. This type of gender-sensitive data can then be transformed into health indicators—an essential foundation of health monitoring and reporting—that can help ensure gender mainstreaming and inform the development of strategies to promote gender equality where needed.^8^,^10^ The use of gender-sensitive health indicators can support the integration of a gender perspective in health monitoring and reporting.^8^ Explanations for gender differences in health and related factors could provide a more nuanced account of health outcomes and risks but are often lacking.^11^,^13^

Conventional health indicators often use sex-stratified data to describe the health situation of women and men, potentially reproducing a binary and cis-normative understanding of sex/gender.^14^ The focus on the gender binary and on cis-gendered individuals—people who identify with the sex they were assigned at birth—neglects the needs and health situation of non-cis-gendered, intersex and non-binary individuals.^13^ The lack of appropriate data and analytical methods poses a challenge to the representation of diversity both between and within gender groups (eg, in terms of age group, ethnicity and socioeconomic status).^9^

In Germany, Federal Health Reporting at the Robert Koch Institute is based on guidelines for the inclusion of a gender perspective in health reporting.^15 16^ This is done through gender-disaggregated reporting of outcomes^17^ and special reports on gender and health.^1 2 18^ However, a set of gender-sensitive health indicators that would consolidate the integration of gender equality into health reporting has not yet been achieved.

Gender-sensitive health indicators aim to shed light on gender-based health inequities and gender inequality as social determinants of health. They are used to measure inequalities in health and health-related outcomes between women, men, gender-diverse individuals and subgroups among them.^9^ Such indicators also relate outcomes and phenomena to underlying sociocultural norms and sociostructural systems of power (eg, gender norms, heteronormativity, racism and ableism). They are important for monitoring whether gender-based health inequalities persist over time.^19^ Gender inequality is a global phenomenon; it is, therefore, important to identify gender-sensitive indicators for health reporting in order to promote gender equality in health.

Incorporating theoretical frameworks and approaches to explain gender-based inequalities in health can lead to a better understanding of health and health-related factors when developing health indicators.^8^ Gender analysis is a theoretical approach that understands gender as a central category of analysis. It aims to identify and address gender inequalities against the background of various individual, social and structural circumstances that affect individuals differently in the gender roles assigned to them.^20^ An intersectionality-informed gender analysis uses gender as the main analytic axis and highlights its intersection with other categories of social differentiation (eg, socioeconomic, sociocultural and sociodemographic variables).^21 22^ Gender as a health determinant affects individuals differently depending on their social location, which is influenced by interlocking systems of privilege and disadvantage (eg, racism, ableism, (hetero) sexism, ageism and classism).^23 24^ In order to enable a more adequate representation of the health situation of certain population groups as well as a better inclusion of societal power relations and structures as health determinants, it is helpful to include intersectionality-informed theoretical approaches.^9^

Scoping reviews are comprehensive and useful tools for rapidly synthesising evidence from a wide range of data sources and research methodologies.^25 26^ For this reason, we aim to conduct a scoping study on gender-sensitive and intersectionality-informed health indicators for health reporting. Within this scoping review, we also aim to identify theoretical frameworks that have been applied or referenced in the development and design of gender-sensitive health indicators. We have checked Prospero and Open Science Framework registries for registered systematic and scoping reviews to ensure our study is new. To our knowledge, this is the first scoping review of gender-sensitive and intersectionality-informed health indicators for health reporting.

## Objectives

The aim of this scoping review is to map the evidence on gender-sensitive and intersectionality-informed indicators for health reporting in order to support the development of a set of indicators for German Federal Health Reporting. In detail, the objectives include the following:

1. To map the current evidence on gender-sensitive health indicators for health reporting.
2. To identify theoretical approaches for gender-sensitive health reporting.
3. To describe the extent to which gender-sensitive health indicators follow intersectionality-informed theoretical approaches.

## Methods and analysis

This scoping review protocol is prepared according to the Preferred Reporting Items for Systematic Review and Meta-Analysis Protocols (PRISMA)^27^ and has been registered at the Open Science Framework (https://doi.org/10.17605/OSF.IO/SHR8M). Potential protocol amendments will be documented in the main manuscript which will follow the PRISMA extension for Scoping Reviews (PRISMA-ScR).^28^ This research is also based on the methodological framework for conducting scoping studies by Arksey and O’Malley^29^ and its enhancement by Levac *et al*^30^ and follows the proposed six stages: (1) identifying the research question, (2) identifying relevant studies, (3) study selection, (4) charting the data, (5) collating, reporting and summarising results and (6) consultation. The completion of the systematic search is scheduled for autumn 2024 and the completion of subsequent analyses for winter 2024. The evidence scoped by means of this review will be incorporated into the development of a gender-sensitive and intersectionality-informed set of health indicators for German Federal Health Reporting. Gender-sensitive and intersectionality-informed health indicators are essential for the promotion of gender equality in health, making them invaluable for health reporting and health information systems globally. Our study is limited to results from other EU-27 and OECD member states. Limiting this scoping review to the EU-27 and the OECD offers advantages in terms of relevance, data quality and manageability of the results, making them more applicable to German Federal Health Reporting. However, it also comes with limitations, especially with regard to the exclusion of a broader global perspective, potentially introducing a bias towards wealthier countries with more developed healthcare systems and disregarding global applicability. We will discuss this limitation in our main manuscript.

### Stage 1: identifying the research question

This scoping study is guided by the following research question, which was jointly developed and refined by the members of the research team (HG, FP and A-CS): What are approaches in health reporting related to health indicators that are considered gender-sensitive and intersectionality-informed and can potentially be adapted to Germany? We understand health indicators as quantitative or qualitative metrics that provide information on topics relevant to public health, including population health, health determinants or health system performance across geographies (eg, countries, regions and communities) or for specific population groups. They provide comparable and actionable information on trends and changes in population health within or between different settings.^31^ Health indicators must meet psychometric requirements for validity and reliability; they must be able to measure changes over time, be easy to use, understandable and ethical.^16 31^ To account for the degree of gender-sensitivity of health indicators, we use a three-category system introduced by the WHO and UNAIDS,^19^ dividing the first category into (a) and (b) to allow for a more nuanced distinction: (1a) gender-specific indicators that refer to a single sex/gender or group of individuals who share certain biological characteristics (eg, the prevalence of prostate cancer or endometriosis) and (1b) gender-related indicators that relate to a sex/gender group (eg, the prevalence of cancers or NCDs for a sex/gender group); (2) gender-disaggregated indicators that measure differences between sex/gender groups in relation to specific metrics (eg, the prevalence of NCDs or cancer by sex/gender and age group, ethnicity or income level); (3) gender-inequality indicators that measure or proxy for gender inequality (eg, indicators that provide plausible links between health outcomes and sociostructural/normative realities such as the gender pay gap, the gender care gap or the prevalence of single parenthood). To determine the presence and extent of intersectionality-informed gender analysis and other frameworks, we will provide a narrative summary of (1) identified intersections between gender and other categories of social differentiation and (2) theoretical and explanatory approaches.

### Stage 2: identifying relevant studies

This scoping review consists of a systematic database search and a targeted search of websites relevant to national and international health reporting. The database search will be conducted in six electronic databases, including Medline (via Ovid), PsycINFO (via Ovid), Embase (via Ovid), Scopus, Cumulated Index to Nursing and Allied Health Literature (CINAHL via EBSCOhost) and Cochrane Library (Cochrane Reviews, Cochrane Protocols, Trials, Editorials, Special Collections and Clinical Answers via Wiley). These databases were selected after careful consideration of their relevance and scope to public health, medicine and the social sciences in addition to feedback from an information specialist. The database searches will be complemented by handsearching reference lists of articles considered relevant during different stages of data screening to identify further relevant references (backward citation search). If an author appears in at least three included full texts, their other publications will be handsearched and evaluated according to our eligibility criteria. The research question was used to operationalise the search strategy into search themes and corresponding English search terms (table 1). Additional MeSH terms and keywords were retrieved using PubMed PubReMiner^32^ for selected, thematically relevant articles. Each search term and combinations of search terms with Boolean and proximity operators were tested individually. These validity tests were documented in a Microsoft Excel spreadsheet (available on request). The search themes were combined with Boolean operators, resulting in the search strategy 1 AND (2 OR 3). The primary search strategy was developed for the Medline database (via Ovid) and peer-reviewed by an information specialist; it will be converted for each database.

**Table 1:** Search themes and search terms

Gender-sensitivity	(((gender* or sex or women* or men) and (sensitiv* or responsiv* or aware* or inclusiv* or equal* or equit* or transform*)) or cisgender* or “cis-gender*” or agender* or nonbinary or “non-binary” or transgender* or “trans-gender*” or transsexual* or “trans-sexual*” or intersex* or “inter-sex*” or intersectionality or (cis adj2 (femme or femmes or masc or mascs or masculine or masculines or people or person or persons or women or woman or men or man or females or males or female or male)) or (trans adj3 (femme or femmes or masc or mascs or masculine or masculines or people or person or persons or individual or individuals or women or woman or men or man or females or males or female or male))).ti,ab. or exp Transgender Persons/ or exp Gender Identity/ or exp Women’s Health/ or exp Men’s Health/ or exp Intersectional Framework/
Health reporting	(((“health information” and (system or systems)) or (health adj (reporting or monitoring or surveillanc*))) and indicator*).ti,ab. or exp Health Information Systems/ or exp Public Health Surveillance/
Health indicators	(health adj indicator*).ti,ab.

In order to obtain a balanced and complete picture of the available evidence, we will conduct a targeted search for grey literature on national (EU-27 and OECD member states) and international websites (eg, WHO, OECD, EU bodies and organisations) relevant to health reporting and health information systems to identify further publications such as reports, working papers, book chapters and official documents.^33^ In addition, we will conduct a Google Search using a combination of search terms on (1) country/setting (eg, “[country]”, “EU”, “European Union”, “OECD”), (2) gender-sensitivity (eg, “gender”, “women”, “men”, “transgender”, “intersex”, “non-binary”) and (3) health indicators (eg, “health indicator”, “health indicators”). These search terms will be translated into the official language of the respective country using DeepL Translate; the search results will be translated into German or English if a language is not covered by the research team. The search terms for the Google Search will be refined iteratively if necessary. We will also include websites (eg, electronic health information systems) as resources if they are relevant to our research question (ie, if they depict gender-sensitive health indicators as defined by us) and no reports can be found. Results retrieved from websites will be reported separately.

To be eligible for inclusion in the scoping study, articles must meet the eligibility criteria formulated on the basis of the PICOS model (Population, Intervention/Exposure, Comparison, Outcome, Setting) applied in this study (table 2). Research articles, reports (including working papers and fact sheets), book chapters and theses will be included. To ensure the scoping review captures the most current developments in the field, we limit our search to research published between 2014 and 2024. This approach may, however, exclude earlier research that continues to be relevant. We will exclude preprints, letters, commentaries, editorials, protocols, conference papers, opinion pieces and the like, as these publication types generally lack the detailed empirical data or comprehensive analyses required for the study’s objectives. Although preprints can provide useful initial insights, their often short format and lack of finalised data may limit their utility for thorough analysis. There are no limitations in terms of language or study design.

**Table 2:** Eligibility criteria

Population	Inclusion: General adult human populationExclusion: Study populations (predominately) under 18 years of age (children, adolescents), non-human populations, analyses of specific population groups (eg, certain employment groups) that do not allow or limit a comparison with the general population
Intervention/Exposure	Inclusion: Health indicators that can be included in health reporting and health information systems, that is, health indicators specifically designed or evaluated for this purposeExclusion: General health indicators that are understood as variables in analyses, health indicators that are not understood as metrics in the context of health monitoring and/or health reporting
Comparison	Inclusion: Health indicators that are reported by or refer to gender: (1a) gender-specific indicators that refer to a single sex/gender or group of individuals who share certain biological characteristics and (1b) gender-related indicators that relate to a sex/gender group; (2) gender-disaggregated indicators that measure differences between sex/gender groups in relation to specific metrics; (3) gender-inequality indicators that measure or proxy for gender inequalityExclusion: Health indicators that are not related to or reported by gender
Outcome	Inclusion: Any type of health or health-related outcomeExclusion: All other non-health related outcomes (eg, policy indicators, biochemical indicators, healthcare management indicators)
Setting	Inclusion: EU-27 and OECDExclusion: Settings other than EU-27 or OECD

EU-27European Union-27OECDOrganisation for Economic Cooperation and Development

### Stage 3: study selection

Study selection will be managed using Rayyan software.^34^ After removing duplicates, studies will be selected iteratively after the titles and abstracts have been screened (stage 1) and the full texts have been checked (stage 2). In both stages, records and full texts will be screened by two reviewers independently using predefined inclusion and exclusion criteria (HG will screen all records, FP and ACS will each screen half of all records). An initial screening of 20% of all records will enable the research team to discuss any uncertainties and refine the inclusion and exclusion criteria if necessary. Disagreements between reviewers at all stages of study selection will be discussed bilaterally and, if needed, with the involvement of a third reviewer until a consensus is reached. An adapted PRISMA flow diagram will be used to describe the review process (online supplemental appendix A).

### Stage 4: charting the data

Full texts that fulfil the inclusion criteria will be extracted for information on study characteristics (authors, publication year, setting, time period, study design/methods), health indicator operationalisation (type/definition, action fields, data source, periodicity, data availability, reference population, scientific background, connections to other indicators), degree of gender-sensitivity (per definition in eligibility criteria), degree of intersectionality-informed gender analysis (definition of intersectionality, intersection(s) to gender, discussion of indicators against the background of power dynamics, references to intersectionality), information on used/referenced theoretical approaches and reported limitations. Multiple publications from a single study will be summarised as one study to avoid overestimating the results. Microsoft Excel spreadsheets will serve as data extraction forms. A data extraction form will be pilot-tested independently by two reviewers who will extract data from a sample of included articles. After discussion and refinement of the data extraction form, one reviewer (HG) will extract data from a full text, and another reviewer will check the data extraction (FP and A-CS will each check one half). The extracted data and discrepancies between the two reviewers will be discussed with the research team.

### Stage 5: collating, summarising and reporting results

The data from the included articles will be synthesised narratively as well as descriptively, and, where appropriate, presented in tables and graphs. Key findings will be analysed and discussed using a narrative method. Reported study limitations and limitations identified by the research team will also be discussed to compensate for the lack of a formal critical appraisal of the studies, as is common in scoping reviews.^28^

### Stage 6: consultation

For the purpose of this study protocol, we consulted with stakeholders recruited from our network of experts from the fields of public health research, health monitoring and reporting. We plan to involve these and other stakeholders in the process of selecting grey literature (ie, identifying relevant websites and documents) and discussing our findings. The results of the scoping review will ultimately inform the development of a gender-sensitive and intersectionality-informed set of health indicators for health reporting. This research is part of the Joint Action Prevent Non-Communicable Diseases (JA PreventNCD, https://www.preventncd.eu/). As part of the JA PreventNCD, this set of health indicators will be evaluated in a focus group discussion with experts from national and international health reporting. A refined set of indicators will be subjected to a Delphi voting process and subsequently included in the German Federal Health Reporting system.

## supplementary material

10.1136/bmjopen-2024-091549 online supplemental file 1

## References

[1] Robert Koch Institute (2020). Gesundheitsberichterstattung des Bundes gemeinsam getragen von RKI und Destatis.

[2] Robert Koch Institute, Gesundheitsberichterstattung des Bundes (2014). Beiträge zur Gesundheitsberichterstattung des Bundes.

[3] Solar O, Irwin A (2010). Social determinants of health discussion. Paper 2 (policy and practice).

[4] Pyone T, Shamoug A, Chan DZX (2021). Gender equality for health and well-being: evaluative evidence of interlinkages with other SDGs. Final report.

[5] Organization for Economic Cooperation and Development (2023). SIGI 2023 global report: gender equality in times of crisis social institutions and gender index.

[6] United Nations Development Programme (2024). Gender inequality index (GII). https://hdr.undp.org/data-center/thematic-composite-indices/gender-inequality-index#/indicies/GII.

[7] European Institute for Gender Equality (2024). Gender equality index. https://eige.europa.eu/gender-equality-index/2023.

[8] Lin V, L’Orange H, Silburn K (2007). Gender-sensitive indicators: Uses and relevance. Int J Public Health.

[9] Pöge K, Rommel A, Mena E (2019). AdvanceGender-Joint project for sex/gender-sensitive and intersectional research and health reporting. Bundesgesundheitsblatt Gesundheitsforschung Gesundheitsschutz.

[10] Sen G, Östlin P, George A (2007). Unequal, unfair, ineffective and inefficient gender inequity in health: why it exists and how we can change it. Final report to the WHO commission on social determinants of health: women and gender equity knowledge network.

[11] Bolte G, Hornberg C, Pauli A, Wede B (2016). Gesundheit – Geschlecht: Eine gesundheitswissenschaftliche Perspektive [Health - Gender: A public health perspective].

[12] Starker A, Rommel A, Saß A-C (2016). Men’s health report - Conclusion and challenges for sex- and gender-sensitive health reporting. Bundesgesundheitsblatt Gesundheitsforschung Gesundheitsschutz.

[13] Rommel A, Pöge K, Krause L (2019). Gender and health in the Federal Health Reporting. Concepts New Challenges. Pub Health Forum.

[14] Pöge K, Rommel A, Starker A (2022). Survey of sex/gender diversity in the GEDA 2019/2020-EHIS study - objectives, procedure and experiences. J Health Monit.

[15] Lange C, Lampert T (2004). Perspectives on gender sensitive health reporting. Gesundheitswesen.

[16] Starke D, Tempel G, Butler J (2019). Good practice in health reporting – guidelines and recommendations 2.0. J Health Mon.

[17] Robert Koch Institute, Robert Koch Institute, Destatis (2015). Gesundheitsberichterstattung des Bundes.

[18] Robert Koch Institute, Robert Koch Institute, Statistisches Bundesamt (2006). Schwerpunktbericht der Gesundheitsberichterstattung des Bundes.

[19] World Health Organization, UNAIDS, Joint United Nations Programme on HIV/AIDS (2016). A tool for strengthening gender-sensitive national hiv and sexual and reproductive health (SRH) monitoring and evaluation systems.

[20] European Institute for Gender Equality (2019). Gender analysis. Gender mainstreaming.

[21] World Health Organization (2020). Incorporating intersectional gender analysis into research on infectious diseases of poverty: a toolkit for health researchers.

[22] Mena E, Stahlmann K, Telkmann K (2023). Intersectionality-Informed Sex/Gender-Sensitivity in Public Health Monitoring and Reporting (PHMR): A Case Study Assessing Stratification on an 'Intersectional Gender-Score.'. Int J Environ Res Public Health.

[23] Iyer A, Sen G, Ostlin P (2008). The intersections of gender and class in health status and health care. Glob Public Health.

[24] Crenshaw K (1989). Demarginalizing the intersection of race and sex: a black feminist critique of antidiscrimination doctrine. Feminist legal theory.

[25] Peters MDJ, Marnie C, Tricco AC (2020). Updated methodological guidance for the conduct of scoping reviews. JBI Evid Synth.

[26] Barnes B, Buchmann M, Mumm R (2022). Evidence syntheses in public health: An overview. Z Evid Fortbild Qual Gesundhwes.

[27] Moher D, Shamseer L, Clarke M (2015). Preferred reporting items for systematic review and meta-analysis protocols (PRISMA-P) 2015 statement. Syst Rev.

[28] Tricco AC, Lillie E, Zarin W (2018). PRISMA Extension for Scoping Reviews (PRISMA-ScR): Checklist and Explanation. Ann Intern Med.

[29] Arksey H, O’Malley L (2005). Scoping studies: towards a methodological framework. Int J Soc Res Methodol.

[30] Levac D, Colquhoun H, O’Brien KK (2010). Scoping studies: advancing the methodology. Impl Sci.

[31] Röding D, Gerlich MG, Walter U (2024). Health indicators. https://leitbegriffe.bzga.de/alphabetisches-verzeichnis/gesundheitsindikatoren/.

[32] Koster J (2014). PubMed PubReMiner. http://hgserver2.amc.nl/cgi-bin/miner/miner2.cgi.

[33] Paez A (2017). Gray literature: An important resource in systematic reviews. J Evid Based Med.

[34] Ouzzani M, Hammady H, Fedorowicz Z (2016). Rayyan-a web and mobile app for systematic reviews. Syst Rev.

