# Supplementary material for: Gender-sensitive and intersectionality-informed health indicators for health reporting: a scoping review protocol
Source: BMJ Open. 2024 Nov 9;14(11):e091549. doi: 10.1136/bmjopen-2024-091549 (PMC11552024; doi:10.1136/bmjopen-2024-091549)
Supplement: online supplemental file 1 [file bmjopen-14-11-s001.pdf]

Supplemental appendix A - PRISMA 2020 flow diagram for new systematic reviews which included searches of databases and other sources

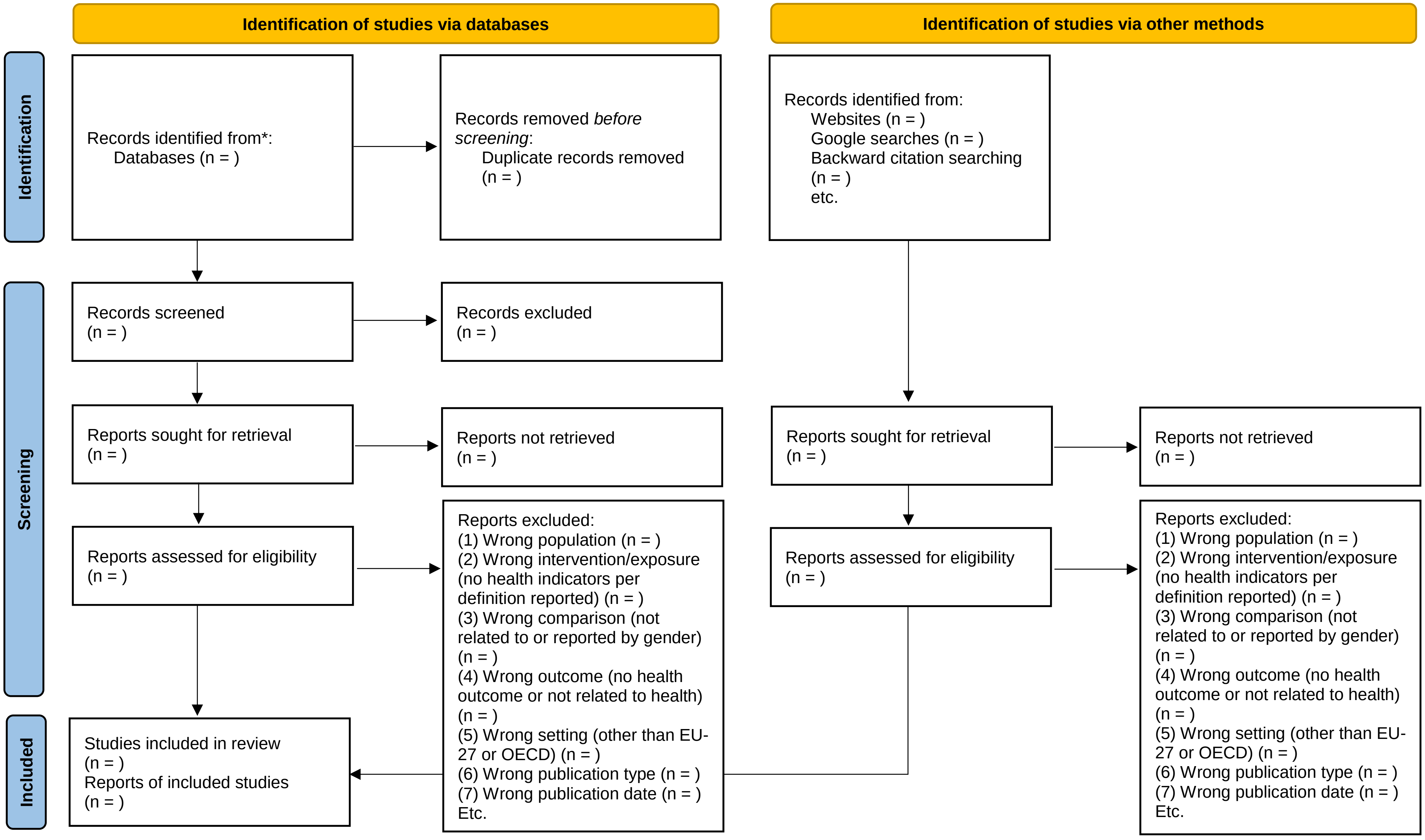

From: Page MJ, McKenzie JE, Bossuyt PM, Boutron I, Hoffmann TC, Mulrow CD, et al. The PRISMA 2020 statement: an updated guideline for reporting systematic reviews. BMJ 2021;372:n71. doi: 10.1136/bmj.n71.
