# Supplementary material for: Gender-sensitive and intersectionality-informed health indicators for health reporting: a scoping review protocol
Source: BMJ Open. 2024 Nov 9;14(11):e091549. doi: 10.1136/bmjopen-2024-091549 (PMC11552024; doi:10.1136/bmjopen-2024-091549)
Supplement: Supplementary file 2 [file bmjopen-2024-091549.reviewer_comments.pdf]

PEER REVIEW HISTORY

BMJ Open publishes all reviews undertaken for accepted manuscripts. Reviewers are asked to complete a checklist review form and are provided with free text boxes to elaborate on their assessment. These free text comments are reproduced below.

ARTICLE DETAILS

Title (Provisional)

Gender-sensitive and intersectionality-informed health indicators for health reporting: a scoping review protocol

Authors

Gencer, Hande; Saß, Anke-Christine; Prütz, Franziska

VERSION 1 - REVIEW

**Reviewer**	**1**
**Name**	**Visintini, Sarah**
**Affiliation**	**University of Ottawa Heart Institute, Berkman Library**
**Date**	**22-Aug-2024**
**COI**	**None to declare**

Thank you for your work on this important topic, and for the opportunity to peer review it. My expertise lies in knowledge synthesis methods generally and search methods in particular, so that is where I will be focusing my efforts in this review.

Line 49: since PRISMA-ScR is a reporting guideline, please differentiate it from your methodological guidance (e.g. and reported according to PRISMA-ScR). You did a good job of this in your methods section.

Lines 48-58: Please provide some more detail about your selection criteria. I think you can probably be more concise about all the searching being done to make room for this.

Lines 197-198: Did the team consider any Gender Studies or LGBTQ+ search resources? For example at my institution we have a database called GenderWatch. That said this is a good selection of health sciences databases!

Line 198: Please specify which Cochrane Library databases as there are 6 (and which platform - Ovid or Wiley). Same for CINAHL (EBSCO I presume?).

Line 200: Did your information specialist write the search? If so are they listed as a co-author? If not please justify this in your response.

Line 201: Please clarify what you mean by "selected articles". Do you mean included studies?

Line 202: I like this approach for forwards citation searching! I will be using this!

Lines 204-212: This section is really wordy. Consider condensing.

Table 1. Thank you for providing your Medline search strategy! Were any existing reviews or search filters consulted in addition to the "pearl growing" technique you describe? If so please cite. You may also want to revisit the MeSH headings used in your search as additional terms were added in the last year under Gender and Sexual Minorities.

Lines 215-219: This section is great but also a bit vague. What sites specifically? These could be listed in a supplemental table.

Lines 248-261: What kind of software will the team use for extraction? Excel?

Line 232: Why will these publication types not be considered for inclusion? Please provide justification.

Line 286: You mention a search peer review here but did not report it in your methods. Please add.

Final Words: This is a very well thought out scoping review project and I appreciate all the work the team is planning to do to supplement the academic literature. Kudos!

**Reviewer**	**2**
**Name**	**Hu, Ran**
**Affiliation**	**The Ohio State University, College of Social Work**
**Date**	**23-Sep-2024**
**COI**	**n/a**

This scoping review protocol is well-written. The authors presented their research question and procedural details clearly and effectively. My suggestion is to provide a rationale for restricting the search timeframe to between 2014 and 2024. if this is primarily due to feasibility concerns, I recommend including this as one of the study limitations.

VERSION 1 - AUTHOR RESPONSE

Dear Editors,

please find attached our point-by-point response to the reviewers' comments.

Dear Editors and Reviewers,

Reviewer: 1

"Thank you for your work on this important topic, and for the opportunity to peer review it. My expertise lies in knowledge synthesis methods generally and search methods in particular, so that is where I will be focusing my efforts in this review."

- Thank you for your thorough, constructive review and the positive feedback!

"Line 49: since PRISMA-ScR is a reporting guideline, please differentiate it from your methodological guidance (e.g. and reported according to PRISMA-ScR). You did a good job of this in your methods section."

- Thank you for pointing this out, this is indeed a very important distinction. We made changes to our abstract accordingly. The sentence now reads: “This scoping review follows Arksey & O’Malley’s methodological framework and its extension by Levac et al. and will be reported according to the PRISMA-ScR extension for scoping reviews.”

"Lines 48-58: Please provide some more detail about your selection criteria. I think you can probably be more concise about all the searching being done to make room for this."

- Thank you for this suggestion. We included information on our selection criteria in the abstract’s second sentence: “We will conduct a database search complemented by a backward citation search using English search terms to find research articles and grey literature (e.g., reports, policy/working papers, book chapters) reporting on gender-sensitive health indicators in the context of health reporting in the EU-27 and OECD published since 2014.”

“Lines 197-198: Did the team consider any Gender Studies or LGBTQ+ search resources? For example at my institution we have a database called GenderWatch. That said this is a good selection of health sciences databases!”

- This is a valid consideration, thank you for this suggestion. We did check sociological and social science databases (e.g. Sociological Abstracts, Social Science Abstracts) but were not able to find relevant publications, as our topic has a stronger public health focus. We believe that we cover all important databases in the field of public health and social epidemiology. We will, however, search websites of organisations and the like with a stronger focus on Gender & Queerness.

"Line 198: Please specify which Cochrane Library databases as there are 6 (and which platform - Ovid or Wiley). Same for CINAHL (EBSCO I presume?)."

- Thank you for pointing this out! We searched all six databases (Cochrane Reviews, Cochrane Protocols, Trials, Editorials, Special Collections and Clinical Answers) on the Cochrane Library website (via Wiley) and for CINAHL we used EBSCOhost. The sentence now reads: “The database search will be conducted in six electronic databases, including Medline (via Ovid), PsycInfo (via Ovid), Embase (via Ovid), Scopus, Cumulated Index to Nursing and Allied Health Literature (CINAHL via EBSCOhost) and Cochrane Library (Cochrane Reviews, Cochrane Protocols, Trials, Editorials, Special Collections and Clinical Answers via Wiley).”

"Line 200: Did your information specialist write the search? If so are they listed as a co-author? If not please justify this in your response."

- The corresponding author wrote the search strategy and developed all the methods; the information specialist did a peer-review of the search strategy and is mentioned in the acknowledgement section.

"Line 201: Please clarify what you mean by "selected articles". Do you mean included studies?"

- Thank you for this comment; we added a clarification: “reference lists of articles considered relevant during different stages of data screening”

"Line 202: I like this approach for forwards citation searching! I will be using this!"

- We are glad to hear this!

"Lines 204-212: This section is really wordy. Consider condensing."

--> Thank you for this feedback! We shortened this section, it now reads: “The research question was used to operationalise the search strategy into search themes and corresponding English search terms (table 1). Additional MeSH-terms and keywords were retrieved using PubMed PubReMiner32 for selected, thematically relevant articles. Each search term and combinations of search terms with Boolean and proximity operators were tested individually. These validity tests were documented in a Microsoft Excel spreadsheet (available upon request). The search themes were combined with Boolean operators, resulting in the search strategy 1 AND (2 OR 3).”

"Table 1. Thank you for providing your Medline search strategy! Were any existing reviews or search filters consulted in addition to the "pearl growing" technique you describe? If so please cite. You may also want to revisit the MeSH headings used in your search as additional terms were added in the last year under Gender and Sexual Minorities."

- We did not use a specific technique other than the ones already mentioned. And thank you for your remark regarding the MeSH headings; we did perform validity tests (https://ovidsp.ovid.com/ovidweb.cgi?T=JS&NEWS=N&PAGE=main&SHAREDSEARCHID=38OhMqryrjrPCt0qZpgEHL0wCm6UiL5Gyxy4lAviF5qSXFR30dzEapYZUFQ2KLMm9) of search terms and MeSH / subject headings (incl. "Sexual and Gender Minorities"[Mesh] / exp "Sexual and Gender Minorities"/), in various combinations. We found that using precise search terms for different gender identities were a much better fit for our purpose compared to including some MeSH / subject headings as the latter yielded too many, rather unprecise hits.

"Lines 215-219: This section is great but also a bit vague. What sites specifically? These could be listed in a supplemental table."

- ➢ We do understand your concerns, however, at this time point, we have not discussed and identified relevant websites. We will provide a supplemental table for the main manuscript, though.

"Lines 248-261: What kind of software will the team use for extraction? Excel?"

- ➢ Thank you for this remark, we will indeed use Excel sheets. We added the following sentence: "Microsoft Excel spreadsheets will serve as data extraction forms."

"Line 232: Why will these publication types not be considered for inclusion? Please provide justification."

- ➢ This is a valid question; we included the following reasoning: "We will exclude pre-prints, letters, commentaries, editorials, protocols, conference papers, opinion pieces and the like, as these publication types generally lack the detailed empirical data or comprehensive analyses required for the study's objectives. Although pre-prints can provide useful initial insights, their often short format and lack of finalised data may limit their utility for thorough analysis."

"Line 286: You mention a search peer review here but did not report it in your methods. Please add."

- ➢ Thank you for pointing this out! We added the following sentence to our methods section (Stage 2): "The primary search strategy was developed for the Medline database (via Ovid) and peer-reviewed by an information specialist; it will be converted for each database."

"Final Words: This is a very well thought out scoping review project and I appreciate all the work the team is planning to do to supplement the academic literature. Kudos!"

- Thank you for your well-thought feedback and the overall review. We appreciate your kind and supporting words!

Reviewer: 2

"This scoping review protocol is well-written. The authors presented their research question and procedural details clearly and effectively. My suggestion is to provide a rationale for restricting the search timeframe to between 2014 and 2024. if this is primarily due to feasibility concerns, I recommend including this as one of the study limitations."

- Thank you for your constructive feedback! We included the following sentence to the relevant section of the manuscript: “To ensure the scoping review captures the most current developments in the field, we limit our search to research published between 2014 and 2024. This approach, may however, exclude earlier research that continues to be relevant.”
